# Coffee and the Risk of Atrial Fibrillation

**DOI:** 10.14740/cr2320

**Published:** 2026-10-24

**Authors:** Shereif H. Rezkalla, Robert A. Kloner

**Affiliations:** a Marshfield Clinic Research Institute, Sanford Health, Marshfield, WI, USA; b Cardiovascular Research Institute, Huntington Medical Research Institutes, Pasadena, CA, USA; c Cardiovascular Division, Department of Medicine, Keck School of Medicine of University of Southern California, Los Angeles, CA, USA

**Keywords:** Caffeine, Coffee, Cardiovascular disease risk, Cardiac arrhythmia

## Abstract

Most hospitals and cardiac rehabilitation facilities provide decaffeinated coffee to cardiac patients in place of regular coffee. Recent studies, however, suggest that coffee drinking may decrease the risk of developing cardiovascular diseases. Review of controlled studies shows that drinking regular coffee in moderation is safe. It may even lead to a decrease in atrial arrhythmias such as atrial fibrillation, and it is safe in patients with ventricular arrhythmias. This is particularly true in patients who drink coffee regularly and in moderation. Energy drinks, however, that contain large doses of caffeine may indeed be harmful and should be avoided. The policies of serving only decaffeinated coffee in hospitals and rehabilitation facilities should be revisited.

## Introduction

Caffeinated drinks such as coffee, tea, and chocolate are commonly used across most countries in the world. Since caffeine is a known stimulant with a slight increase in heart rate and blood pressure, it is used with some caution in cardiac patients. On cardiac floors in most hospitals, decaffeinated coffee is preferred to regular coffee for most patients. No clear studies, however, suggest obvious harm from moderate coffee drinking [1]. On the contrary, moderate consumption of regular coffee is associated with a decreased risk of diabetes [2], stroke [3], and the risk of developing cardiovascular disease [4]. The suggested benefit included a mortality benefit. Drinking three to four cups of regular coffee per day decreases both cardiovascular as well as total death [5].

In this report, we will focus on the effect of drinking regular coffee on developing cardiac arrhythmia including atrial and ventricular arrhythmias. Special attention will focus on atrial fibrillation (AF) being one of the common asymptomatic cardiac irregularities.

## Methods

We performed a search utilizing PubMed from the years 2000 to 2026. Keywords were coffee and cardiac arrhythmias. A total of 135 articles were identified. All abstracts were reviewed, and relevant articles were critically reviewed and were the basis for this review.

## Coffee and AF

A variety of atrial and ventricular arrhythmias are linked to coffee drinking. The two most clinically relevant arrhythmias are AF and ventricular tachycardia. This brief report will focus on both.

Marcus et al [6] conducted a prospective randomized study to examine the effect of acute regular coffee drinking on 100 healthy volunteers. The entire cohort was randomly assigned to drink coffee or to avoid any caffeine drinking, including decaffeinated coffee, for two consecutive days by computerized random assignment. Cardiac arrhythmias were observed by continuous electrocardiographic monitoring. The primary endpoint was premature atrial contractions during coffee drinking and caffeine avoidance. The coffee drinking period was associated with 58 beats per day, while caffeine avoidance resulted in 52 beats per day (non-significant difference). There was no statistically significant difference in other arrhythmias, including premature ventricular complexes and non-sustained ventricular contractions [6]. A more recently published study, a prospective randomized clinical trial targeted patients who are known coffee drinkers, with planned electro-cardioversion. Following the cardioversion, patients were randomized to coffee consumption of at least one cup of regular coffee per day or complete avoidance of consumption of caffeine. Patients were then prospectively followed for 6 months. Not only did the regular coffee drinking group have no higher rate of recurrent AF, but indeed, caffeine drinking was protective from recurrent AF. The coffee drinking group had a recurrence of AF of 47%, while the control group had a recurrence rate of 64% (P value of 0.01) [7]. Therefore, in controlled randomized studies, regular coffee did not increase cardiac arrhythmias in both healthy people and in those patients at risk of having recurrent AF.

A seminal study on the effect of caffeinated coffee on cardiac arrhythmias was reported from a United Kingdom biobank. A total of 286,258 patients with no previous history of cardiac arrhythmias were prospectively followed for 12 years, between 2006 and 2018. The primary endpoint was development of cardiac arrhythmias and its relation to drinking caffeinated coffee. There was no statistically significant difference in ventricular premature beats or ventricular tachycardia between drinking regular coffee or avoiding all caffeine products. What was surprising was that drinking regular coffee was protective for development of new AF or supraventricular arrhythmias with every single cup of regular coffee being associated with a 3% lower risk of supra-ventricular tachycardia and AF [8]. Multiple other studies [9–15], mostly prospective with long-term follow-up, conducted either in healthy individuals with development of new-onset AF or in patients with paroxysmal AF with recurrence of the arrhythmia, are summarized in Table 1. No study showed any increase in the incidence of AF in people who drank regular coffee. Indeed, some studies reported a decrease in the incidence of AF in the regular coffee drinking group compared to the control population who avoided coffee and caffeinated products.

**Table 1:** Controlled Studies Investigating the Effect of Coffee Drinking on the Incidence of Atrial Fibrillation and Other Cardiac Arrythmias

Study	Year	Design	# Patients	Population	Control	Outcome
Wong et al [7]	2025	Prospective open label	200; followed for 3 years	AF, followed after cardioversion	Non-coffee drinkers	Coffee drinkers had less recurrence of AF
Marcus et al [6]	2023	Prospective, randomized	100; followed for 14 days	Healthy patients evaluated for number of premature atrial contractions	Non-coffee drinkers	Consumption of coffee did not result in higher frequency of premature atrial contractions by continuous monitoring
Iten et al [9]	2024	Prospective observational	3,835; followed for 4–7 years	Patients with documented AF	Non-coffee drinkers	Patients with persistent AF had reduced risk of acute heart failure and lower mortality in coffee drinkers
Kim et al [8]	2021	Prospective cohort from UK Biobank	386,258 patients who developed cardiac arrhythmias in 4–5 years	Patients who developed cardiac arrhythmias and drank coffee		Each cup of coffee had a 3% lower risk of cardiac arrhythmias, including supraventricular and ventricular arrhythmia
Bazal et al [10]	2021	Prospective study in Spain	254,462 patients at high risk of cardiovascular disease	Patients who developed new AF and were coffee drinkers	Patients who developed more AF but were non-coffee drinkers	Moderate coffee drinking led to reduction of AF
Yuan et al [11]	2019	Data from AF Consortium	588,190	Patients who had AF and were coffee drinkers	Patients who had AF but did not drink coffee	Coffee drinking showed no cardiac nor any effect on the risk of AF
Bodar et al [12]	2019	Prospective study of Physicians’ Health Study	18,960; follow-up for 9 years	New cases of AF in the population	No consumption of coffee	Lower risk of AF in people who consumed 1–3 cups of coffee per day
Mostofsky et al [13]	2016	Population-based study	57,053; follow-up for 13.5 years	Patients from Danish diet study who reported AF	Non-coffee drinkers	Higher level of coffee consumption had lower rate of incidence of AF
Larson et al [14]	2015	12 years of follow-up	76,475; follow-up for 12 years	Patients from the Swedish population	Non-coffee drinkers	No evidence that coffee increased the risk of incidence of AF
Mattioli et al [15]	2009	48 h after detection	600 patients with first episode of AF	Hypotensive patients with first know AF	Coffee vs. non-coffee consumption	Coffee consumption led to less likely spontaneous conversion of AF

AF: atrial fibrillation.

Caldeira et al [16] performed a meta-analysis of seven studies investigating the effect of regular coffee on the incidence of AF. Studies evaluated 115,993 patients during the period of 2001 until 2012. Caffeine exposure was not associated with any increase in the risk of AF. Indeed, low-dose caffeine exposure resulted in a 13% reduction in that incidence.

Caffeine, the most prominent component of coffee is 1,3,7-trimethylxanthine, the chemical name for caffeine. It has many biologic effects on the body, most prominently the release of norepinephrine and sensitization of dopamine receptors. As a natural central nervous system stimulant, caffeine works by blocking adenosine receptors in the brain to prevent drowsiness, enhance alertness, and boost physical performance. Acute administration of regular coffee leads to a rise in blood pressure, particularly in people who do not consume caffeine regularly [17]. In patients who have regular consumption of a moderate amount of coffee, this effect is attenuated, and it was suggested that caffeine metabolites may reduce the incidence of hypertension. Chen et al [18] analyzed data from the National Health and Nutrition Survey in 2018. A total of 6,076 elderly patients were followed for about 7 years. Moderate intake did not significantly affect blood pressure. Coffee drinking, however, resulted in reduced total mortality and a reduction in cardiovascular mortality [18]. Thus, although the main reason doctors in cardiac units avoid use of regular coffee is concern about arrhythmia, this practice does not appear to be convincing in the current controlled studies.

## Pathophysiology of Caffeine Intake

Caffeine decreases systemic inflammation and reduces oxidative stress; both may increase the incidence of AF. Most importantly, caffeine leads to blockade of adenosine receptors [19], which will result in prolongation of the atrial refractory period, an environment that decreases development of AF.

## Ventricular Arrhythmias

Graboys et al [20] evaluated the caffeine effect on 50 consecutive patients with ventricular arrhythmias. It included patients with non-sustained ventricular tachycardia, recurrent ventricular tachycardia, and ventricular fibrillation. It included continuous electrocardiographic monitoring of patients randomly assigned to decaffeinated coffee or decaffeinated coffee plus 200 mg of caffeine. There was no difference in ventricular ectopy during bicycle exercise test.

In 35 patients with recent myocardial infarction, continuous electrocardiography was performed. Patients were randomized to 450 mg of caffeine daily or placebo. There was no increase in ventricular ectopy following caffeine ingestion [21].

In the determinants of myocardial infarction onset study, patients with acute myocardial infarction were examined to see the effect of coffee intake on ventricular ectopy [22]. There was no increase in ventricular ectopy following one or two cups of coffee. There was, however, a slight increase in ectopy following more than two cups of coffee per day.

Chelsky et al [23] conducted a prospective study on 22 patients with symptomatic non-sustained ventricular tachycardia. Patients underwent an electrophysiologic study at baseline and at 1 h following a 275 mg ingestion of caffeine. Caffeine did not alter the inducibility or severity of ventricular arrhythmias.

Administration of a 250 mg dose of caffeine to healthy non-caffeine users increased plasma adrenaline and noradrenaline. This may be the reason cardiac units restrict the use of regular coffee. In people who drink coffee regularly, there is complete tolerance to the effect of catecholamine rise, and in only a few days, the consumption of coffee does not result in any rise in adrenaline or noradrenaline [24]. In the QACC/AHA guidelines for management of ST-elevation myocardial infarction, it is suggested that patients who are regular coffee drinkers may drink coffee, provided they restrict the consumption to only one cup per day [25].

## Clinical Recommendation

Because of laboratory studies and some earlier case reports that suggest catecholamine release following caffeine intake, caution in providing regular coffee in cardiac units and in some cardiac patients was adopted. Multiple studies have shown that use of regular coffee in small to moderate amounts in cardiac patients is safe. This is likely true in patients who are regular coffee drinkers and the use is limited to one or two cups per day. Patients who consume coffee on a regular basis develop tolerance to caffeine, with little or no catecholamine release. This recommendation is limited to coffee and is not extended to other caffeinated drinks, such as energy drinks. Of note, there are no safety studies that have investigated the effect of decaffeinated coffee on cardiac patients. A recent scientific statement from the American Heart Association Council on Lifestyle and Cardiometabolic Health presented a comprehensive review about beverages and foods with caffeine. It is suggested that habitual coffee consumption is associated with a lower risk of type 2 diabetes and may be associated with lower risk of coronary artery disease and heart failure. In their report, regular filtered coffee was associated with a lower risk of developing AF [26]. There is a need to revisit the use of regular coffee in cardiac patients admitted to cardiac units.

## Conclusion

Most hospitals provide decaffeinated coffee to cardiac patients in place of regular coffee. The concern is coffee may cause catecholamine release, which increases the frequency of atrial arrhythmias such as AF, or ventricular arrhythmias such as ventricular tachycardia. A majority of published studies have shown that regular coffee consumption is safe, provided that patients are habitual coffee drinkers and the intake is limited to a small or moderate amount. Patients who are not habitual coffee drinkers or have history of palpitations following coffee drinking may consider avoiding regular coffee. Not enough data are available for patients with acute coronary syndromes, including ST-elevation myocardial infarction and non-ST-elevation myocardial infarction. Until there are sufficient data, those patients should avoid caffeinated coffee. Regular coffee use in hospitals and cardiac rehab units should be reviewed for more proper recommendations.

## Data Availability

The authors declare that data supporting the findings of this review are available within the article.
